# Cytokine profile of pediatric patients with obsessive-compulsive and/or movement disorder symptoms: A review

**DOI:** 10.3389/fped.2022.893815

**Published:** 2022-08-19

**Authors:** Rebecca Alison Fabricius, Camilla Birgitte Sørensen, Liselotte Skov, Nanette Mol Debes

**Affiliations:** ^1^Department of Pediatrics and Adolescent Medicine, Copenhagen University Hospital, Herlev, Denmark; ^2^Department of Clinical Medicine, University of Copenhagen, Copenhagen, Denmark

**Keywords:** cytokines, autoimmune, pro-inflammatory, obsessive-compulsive, movement disorders

## Abstract

Cytokines are an important modulator of the immune system and have been found to be altered significantly in many neurological and psychiatric disorders, like obsessive compulsive disorder (OCD) and movement disorders. Also, in pediatric autoimmune neuropsychiatric disorders associated with group A streptococcal infections (PANDAS), which are characterized by abrupt debut of symptoms of OCD and /or movement disorder symptoms, alterations in the immune system have been suggested. The aim of this paper was to review the current literature on the cytokine profile of pediatric patients with symptoms of OCD and/or movement disorder symptoms. A search of PubMed and Medline was performed with specific keywords to review studies measuring cytokines in pediatric patients with symptoms of OCD and/or movement disorders. Nineteen studies were found, twelve of which included a healthy control group, while four studies had control groups of children with other disorders, primarily neurological or psychiatric. One study compared cytokines measurements to reference intervals, and two studies had a longitudinal design. Many cytokines were found to have significant changes in patients with symptoms of OCD and/or movement disorders compared to both healthy controls and other control groups. Furthermore, differences were found when comparing cytokines in periods of exacerbation with periods of remission of symptoms in study participants. The cytokines that most studies with healthy control groups found to be significantly altered were TNF-α, IL-1β and IL-17. Although the exact role of these cytokines in OCD and movement disorder symptoms remains unclear, the available literature suggests a proinflammatory cytokine profile. This offers interesting perspectives on the pathogenesis of OCD and/or movement disorder symptoms in children, and further research into the implications of cytokines in neuropsychiatric disorders is warranted.

## Introduction

Autoimmunity is the failure of the immune system to recognize the organism as itself. The classic component of autoimmune disorder is the inflammation (1), which is a normal physiological defense against infection and tissue damage. However, in many autoimmune disorders an abnormal inflammatory response is associated with tissue and organ damage (2). Autoimmunity can be induced through many different mechanisms. One common etiology is post-infectious, as is seen in Guillain Barré Syndrome, rheumatic fever, and glomerulonephritis. Although many pathogens can cause autoimmunity, group A streptococci (GAS) is especially potent (3). Many autoimmune diseases, for example systemic lupus erythematosus, have comorbid psychiatric symptoms, suggesting a connection between disorders of the immune system and psychiatric disorders (1). Major depressive disorder has been studied extensively. In patients suffering from depression cardinal features of inflammation, such as elevated cytokines in peripheral blood and cerebrospinal fluid (CSF) as well as other acute inflammatory mediators, have been seen (4). In other psychiatric disorders such as obsessive-compulsive disorder (OCD), chronic tic disorder (CTD) and Tourette's Syndrome (TS) it has also been suggested that a subgroup of patients might have immune-related and/or post-infectious autoimmune etiology (5).

Historically, several studies have described patients with OCD and/or movement disorder after infections. In 1978, Kondo and Kabasawa reported a sudden and abrupt debut of a tic disorder after fever in a 11 year- old boy who had elevated antistreptolysin antibodies and responded well to treatment with corticosteroids (6). In the 1980s and 1990s, patients with OCD symptoms developing simultaneously with Sydenham's Chorea (SC) related to GAS infections were described (5). In 1990, children with movement disorders were found to have elevated antistreptococcal titers, and a link between an antecedent GAS infection and movement disorders was suggested (7). In 1995, Allen et al. (8) reported four cases of abrupt, severe onset or a worsening of OCD and/or movement disorder in form of tics. All patients had had recent infections, GAS or viral, and the essential symptoms were determined to be pediatric, infection-triggered, autoimmune neuropsychiatric disorders (PITANDS) (8). Pediatric autoimmune neuropsychiatric disorders associated with streptococcal (group A) infections (PANDAS) were first described in 1998 by Swedo et al. (9) and were described as presence of OCD and/or a tic disorder temporally associated with a GAS infection.

The role of cytokines in neuroinflammation and as pathophysiological mechanism in psychiatric disorders is of interest. Cytokines are small glycoproteins which can be produced by many different cells in all organs. They play an important role in brain development and promotion of normal brain function (10) and can, amongst many other things, create or hinder inflammation and recruit cellular components of the immune system (11). However, they can turn detrimental for the brain if strongly activated by infection or injury, as high levels of pro-inflammatory cytokines can negatively impact memory, neural plasticity and neurogenesis (10).

However, not much is known about the cytokine profile in children with neuropsychiatric symptoms. An improved understanding of the cytokine profile of these patients could offer insight into the pathogenesis of these disorders. In this article we review the available literature, to determine the cytokine profile of children with neuropsychiatric symptoms as seen in OCD, TS, SC, CTD, PANS and PANDAS.

## Method

In the present review, a literature search in the Pubmed, PMC and MEDLINE databases was performed. The initial search with the following terms; {[PANDAS (Body-Key Terms)] OR [PANS (Body-Key Terms)] OR [OCD (Body-Key Terms)] OR [Sydenham's chorea (Body-Key Terms)] OR [Tourette's disorder (Body-Key Terms)]} AND {[cytokine (Body-Key Terms)] OR [immune(Body-Key Terms)]} was conducted through the PubMed Central (PMC) database and yielded 342 results. A supplementary search with the terms “(cytokine) AND [(OCD) OR (PANDAS) OR (PANS) OR (Tourette's disorder) OR (tics) OR (Sydenham's chorea)] AND [(pediatric) or (children)]” was carried out on Pubmed.gov database, which includes PMC and MEDLINE, to see if any supplementary materials not found in the original search could be added. This yielded 90 results.

A total of 432 article titles and abstracts were assessed for relevance for the review. Exclusion criteria were studies written in other languages than English, letters to the editor, conference presentations, editorials, comments, or opinions. Seventy articles were included for close reading of the full text. Furthermore, 52 articles of potential interest were added through the references of the aforementioned articles.

In total, 122 article abstracts were systematically read to clarify if the articles documented any kind of cytokine measurement in subjects with either PANDAS, PANS, OCD, TS or SC, and 33 articles were identified. From these 33 articles, 19 had pediatric populations and 14 had only adult populations and were therefore excluded.

## Results

Of the 19 articles describing cytokines in pediatric patients with obsessive/compulsive symptoms and/or movement disorder symptoms, 12 had included a healthy control group, 5 had control groups with other disorders or no control group and 2 had a longitudinal study design.

The studies examining cytokines in pediatric patients compared to healthy controls are summarized in Table 1. The studies primarily used ELISA but also other immune assays to examine the different cytokine levels in primarily serum or plasma. The studies subjects differed in included number, ages, diagnosis and in extent of use of psychotropic medication. The cytokines the studies have chosen to examine also differed between studies, however many studies chose to examine TNF-a, IL-6, IL-1b, IL-2, IL-17A and IL-12, and many of the studies found significant alterations of these cytokines when compared to healthy controls. The cytokines with significant results from Table 1 are summarized in Table 2.

**Table 1:** Studies examining cytokines in pediatric patients with obsessive-compulsive and/or tic symptoms compared to healthy controls.

**Article**	**Diagnosis**	**No of patients (no of healthy controls)**	**Medication**	**Tissue tested**	**Study design**	**Cytokines tested**	**Significant results**
Bos-Veneman et al. (12)	TS or CTD	66 (71)	56% of patients used psychotropic medication, antipsychotic primarily	Serum	Cytokines measured with multiplex cytokine array, read on luminex platform	IL-2, IL-4, IL-5, IL-12, sIL-2R, TNF-α, IFN-γ, sVCAM-1 and sICAM-1	No differences found, but lower frequency of detectable IFN-g levels in patients, medication had no association with levels of cytokines
Cheng et al. (13)	TS	40 (40)	Unknown how many patients received any medication	Plasma	Cytokines measured using solid phase sandwich ELISA	IL-6, sIL-6R, IL-1β, sgp130 and IL-17	IL-1β, IL-6, IL-17 and sgp130 were increased, and sIL-6R was decreased. Also found higher proportion IL-6 positive lymphocytes and IL-17 positive lymphocytes
Çolak Sivri et al. (14)	OCD, no coexisting tics	44 (40)	Patients were psychiatric medication naive, apart from 5 who had a history of medication but were unmedicated at time of study	Serum	Cytokines and chemokines measured using ELISA	IL-12, IL-17, TGF-β, TNF-α, sTNFR1, sTNFR2, IL-1β, CCL3, CCL,24, CXCL8, BDNF	TNF-α higher for OCD, while IL-12 lower
Gabbay et al. (15)	TS (+/- OCD)	32 (16)	22% patients were medication naive, 78% were taking psychotropic medication at assessment	Plasma	Cytokines measured using ELISA, except for TNF-α, IL-1β, IL-12, IL-6 and IL-2 here specific high sensitivity human quantikine assays were used	TNF-α, IL-12, IL-β, IL-6 and IL-2	TS+OCD subgroup elevated IL-12 compared to control and IL-2 increased in TS+OCD compared to TS-OCD. No changes when adjusted for psychotropic medication
Gariup et al. (16)	OCD and tics	8 in OCD and tics group (34)	All patients received some form of medication	Serum	Cytokines measured with Luminex ultra-sensitive kit, except IP-10 and MCP-1 were measured with ELISA	IL-1β, IL-2, IL-4, IL-5, IL-6, IL-8, IL 10, GM-CSF, IFN-γ, TNF-α, IFN-γ-IP-10, MCP-1	IL-1β and IL-8, IP-10 higher for OCD and tics
Leckman et al. (17)	OCD (+/- PANDAS)	46 (31)	Majority of patients were receiving medication to control tics and/or OCD symptoms	Serum	Cytokines measured at study entry and exacerbations with multiplex ELISA	IL-2, IL-4, IL-5, IL-6, IL-10, IL-12, INF-α, INF-γ, TNF-α	TNF-α and IL-12 levels higher in patients, non-PANDAS more likely than PANDAS to have elevated TNF-α. Patients not receiving medication had highest baseline level of IL-12 and TNF-α. IL-5 was higher for non-medicated than medicated and controls
Li et al. (18)	TS	58 (30)	Treatment experience including dopaminergic receptor antagonists was exclusion criteria	Serum	ytokines measured with ELISA	IL-6, IL-8, and TNF-α	Decrease in levels of both IL-6 and IL-8 and increase in the level of TNF-α
Matz et al. (19)	TS	46 (43)	65 % of patients received psychotropic medication	Serum	Cytokines measured with Bio-Plex cytokine assay, except for IL1-ra and CD14 a specific quantikine Immunoassay was used for each	TNF-α, IL-6, CD14 and IL1-ra	TNF-α, IL1-ra and CD14 lower for TS children (unclear how many had comorbid OCD). No differences between medicated and non-medicated patients.
Pranzatelli et al. (20)	TS with streptococcus markers	5 (26)	Patients were one week off medication when examined, all were medicated	Serum and CSF	Cytokines measured with ELISA	Intracellular IFN-γ and IL-4, CXCL13, CXCL10, CCL19, CCL21 and CCL22	No differences found.
Rodriguez et al. (21)	OCD	102 (47)	80% of patients were medicated	Monocytes	LPS stimulated cytokines measured with multiplex luminex assay	IL-1β, IL-6, GM-CSF, TNF-α and IL-8	Higher production of IL-1β, IL-6, GM-CSF, TNF-α and IL-8. Levels were higher for unmedicated patients than medicated, which were higher than controls
Simşek et al. (22)	OCD	34 (34)	Psychotropics were exclusion criteria, unclear if all were medication naive	Serum	Cytokines were measured with BD cytometric Bead Array analysis	IL-2, IL-4, IL-6, IL-10, IL-17A, IFN-γ and TNF-α	Patients had increase in IL-2, TNF-α and IL-17A
Yeon et al. (23)	TS	26	62% of patients were unmedicated	Serum	Cytokines were measured using ELISA	MCP-1, IL-1β, IL-17A, IL-6, IL-12p70, and TNF-α	IL-17A, IL-12p70, IL-6 and TNF-α were increased in patients. TNFα was found to increase in unmedicated patients compared to patients taking medication

BDNF, brain derived neurotrophic factor; CCL, CC motif chemokine ligand; CD, cluster of differentiation; CSF, cerebrospinal fluid; CTD, chronic tic disorder; CXCL, C-X-C motif chemokine ligand; ELISA, enzyme linked immunosorbent assay; GM-CSF, granulocyte-macrophage colony-stimulating factor; IFN, interferon; IL, interleukin; IL1-ra, IL-1 receptor antagonist; IP, interferon gamma-induced protein; LPS, lipopolysaccharide; MCP, membrane cofactor protein; OCD, obsessive-compulsive disorder; PANDAS, pediatric autoimmune neuropsychiatric disorder associated with streptococcal infections; sgp, soluble glycoprotein; sI/VCAM, soluble intercellular/vascular cell adhesion molecule; sTNFR, soluble TNF receptor; TNF, tumor necrosis factor; TS, Tourette syndrome.

**Table 2:** Overview of cytokine changes in studies in pediatric patients with tics and/or obsessive-compulsive symptoms compared to healthy controls.

**Cytokine**	**Increase**	**Decrease**	**No differences**
TNF-α	Çolak Sivri et al. (14), Leckman et al. (17), Rodriguez et al. (21), Simşek et al. (22), Li et al. (18), and Yeon et al. (23)	Matz et al. (19)	Bos-Veneman et al. (12) and Gariup et al. (16)
IL-6	Rodriguez et al. (21), Cheng et al. (13), and Yeon et al. (23)	Li et al. (18)	Leckman et al. (17) and Simşek et al. (22)
IL-1β	Gariup et al. (16), Rodriguez et al. (21), and Cheng et al. (13)		Çolak Sivri et al. (14) and Yeon et al. (23)
IL-2	Gabbay et al. (15) and Simşek et al. (22)		Bos-Veneman et al. (12), Gariup et al. (16), and Leckman et al. (17)
IL-17(A)	Cheng et al. (13), Simşek et al. (22), and Yeon et al. (23)		Çolak Sivri et al. (14)
IL-12	Gabbay et al. (15) and Leckman et al. (17)	Çolak Sivri et al. (14)	Bos-Veneman et al. (12)
IL-8	Gariup et al. (16) and Rodriguez et al. (21)	Li et al. (18)	
GM-CSF	Rodriguez et al. (21)		Gariup et al. (16)
IP-10	Gariup et al. (16)		
IL-12p70	Yeon et al. (23)		
IL1-ra		Matz et al. (19)	
CD-14		Matz et al. (19)	
sgp130	Cheng et al. (13)		
sIL-6R		Cheng et al. (13)	

CD, cluster of differentiation; GM-CSF, granulocyte-macrophage colony-stimulating factor; IP, interferon gamma-induced protein; IL, interleukin; IL1-ra, IL-1 receptor antagonist; sgp, soluble glycoprotein; sIL-6R, soluble IL-6 receptor, TNF, tumor necrosis factor.

Five studies have included controls with other disorders, other neurological diseases, patients undergoing tonsillectomy (24–28). In general, these studies have included less participants (*N* = 12–24) and they tested a broad range of cytokines through primarily ELISA. The medication status was not always described. The significant results included a higher level of IL-2 in patients with OCD compared to controls with ADHD or schizophrenia (24). Elevated IL-6 and IL-17A in D2R specific T-cells from subset of patients with SC, TS or PANS were seen compared to controls with neurological disease (25). Compared with children with non-inflammatory neurological diseases like epilepsy, IL-4 was found to be increased in patients with acute and persistent SC and Il-10 and IL-12 were only elevated in patients with acute SC (26). Another study analyzed cytokines in tonsils from PANDAS children compared to children undergoing tonsillectomy for either obstructive sleep apnea or chronic tonsilitis (29). A significant increase of TNF-a and eoxtaxin-3 was found in patients with PANDAS, while IL-8, IP-10, IL-17A IL-10 and IL-12 were significantly decreased (27). Another study compared serum cytokines from PANS children to standard reference values and found them to be within the normal reference (28).

Two studies had a longitudinal design (30, 31). The first study investigated children with TS and CTD with or without OCD and compared periods of exacerbation of symptoms to periods of remission. TNF-α was higher in exacerbation compared to remission. They found no differences in serum cytokine levels between tic-OCD patients and tic+OCD patients (30). The second longitudinal study compared PANDAS debut to periods of exacerbations and no significant differences in cytokines were found (31).

## Discussion

The aim of this study was to review the cytokine profile of pediatric patients with neuropsychiatric symptoms as seen in OCD, TS, SC, PANDAS, PANS or CTD. We found that cytokines for these patient groups appear to be affected in a proinflammatory direction. Of special interest were TNF-α, IL-17 and IL-1β, as most studies measuring these cytokines found a significant increase compared to healthy controls. The studies that had included healthy controls found significant increases in especially the cytokines TNF-α, IL-17 and IL-1β, but many other cytokines were also reported as being significantly increased or decreased in patients compared to healthy controls, as shown in Table 2. The studies with other control groups had more heterogenous results, most likely due to smaller sample sizes and more heterogeneity in the control groups. Only two cytokines were reported significantly altered in more than one study with a non-healthy control group (Il-17 and IL-10), however these were reported as significantly increased in one study and significantly decreased in the other. The studies with longitudinal design were also challenged by their sample size. One study found significantly increased TNF-α in periods of exacerbations, while the other did not.

TNF-α is a proinflammatory cytokine, as it can initiate a strong inflammatory response in nucleated cells, but it can also act as an immunosuppressive mediator by limiting the inflammatory responses. Furthermore, it has a role in inhibiting the development of autoimmune diseases (32). TNF-α has been found to be of importance in many neurological and psychiatric disorders. A recent study found that maternal OCD was related to a significantly higher level of cord blood TNF-α which also was positively correlated with maternal anxiety level (33). Some polymorphisms of the TNF-α gene have found to be associated to OCD susceptibility (29, 34) and others to TS (35). In this review, increased levels of TNF-α were seen across different diagnoses. Significantly increased levels were seen in two studies with patients with TS (18, 23) and in three studies with patients with OCD (14, 21, 22). Furthermore, one longitudinal study found that TNF-α levels were increased in periods of exacerbations in children with TS/CTD (30). Although these findings not have been replicated by other studies (12, 19, 31), they do suggest that TNF-α might be involved in children with obsessive-compulsive and/or tic-related symptoms. Based on the findings in this review, we suggest that a dysregulation or increase of TNF-α, perhaps on a genetic basis, could be associated with obsessive-compulsive and tic symptoms.

IL-1β is also a proinflammatory cytokine, and has been of interest in various central nervous system (CNS) diseases, like multiple sclerosis (36). In children with febrile seizures, elevated levels of IL-1β (as well as TNF-α) in CSF were seen (37). In a meta-analysis, an association was found between the risk of febrile seizures and epilepsy and polymorphism in the IL-1β (511) gene (38). IL-1β has been found to be associated with Post-Traumatic Stress Disorder and bipolar disorder (39, 40). However, the literature on IL-1β and its association to obsessive-compulsive and/or tic symptoms is scarce. One systematic review and meta-analysis found significant reduction in IL-1β compared to healthy controls; importantly this was almost exclusively based on data from adults (41). In a Chinese population with OCD no association was found in IL-1β−511 polymorphism compared to healthy controls (42).

IL-17 (often also called IL-17A) is a proinflammatory cytokine and is mainly expressed by CD4+ TH17 cells (32). In CNS, IL-17 is a mediator between immune cells and tissue, and it was found that an artificial overexpression of IL-17 activates glial cells and enhances neuroinflammation (43). IL-17 has been reported to synergize with other proinflammatory cytokines, such as TNF-α, and potentiate their effects (44, 45). IL-17 has also been reported to be associated to different neurological and psychiatric disorders. In Parkinson's Disease patients, increased levels of IL-17 were correlated with higher levels of anxiety and depression (46). Depression has also been associated with IL-17. One study found IL-17 to be significantly increased in peripheral blood in depressive patients compared to healthy controls (47), although a correlation between severity of depression and IL-17 levels was not found (48). In children with autism, IL-17 levels were elevated compared to healthy controls, and were significantly correlated with the severity of autism (49). Although dysregulation of IL-17 has been found to be triggering several autoimmune diseases in murine models (50), literature on its role in obsessive-compulsive or tic symptoms remains sparse (41). We suggest therefore that studies investigating cytokines in children with obsessive-compulsive and/or tic symptoms in the future should include this cytokine in order to elucidate its role.

An important consideration regarding the methods used in the included articles is that most of the studies measured peripheral cytokines, in serum, or plasma, or tonsil tissue. It can be argued that peripheral cytokines are unreliable surrogate markers of the cytokines in the CNS, as peripheral cytokines can be influenced by many other variables such as age, body mass index, medication, smoking, stress and circadian fluctuation (51). On the other hand, it is important to recognize that the blood-brain barrier (BBB), initially giving reason for the immune privilege hypothesis, can be impaired in various ways, for example by inflammatory cytokines which appear to play a crucial role in allowing antibodies to cross the BBB by impacting (52) stability of the BBB (53). TNF-α induces formation of gaps in BBB by internalizing tight junction protein *via* upregulation of nuclear factor kappa-light-chain-enhancer of activated B cells (NF κB) and its transcription myosin light chain kinase (54, 55). IL-17 also disrupts the BBB tight junctions and promotes the transmigration of CD4+ lymphocytes through TH17 cells' ability (activated by IL-17) to permeabilize the BBB (56). Peripheral inflammation has also been reported to affect the BBB permeability in other psychiatric disorders (schizophrenia, bipolar disorder and major depressive disorder) (57).

The included studies have used various immune assays for measuring cytokines, performed on various biological materials (serum, plasma, CSF and tonsil tissue) from differing patient groups (TS, OCD, SC, CTD, PANS and PANDAS with different comorbid combinations). Only some of the studies reported on medication status, and not all of them included medication status as confounder. These considerations, and the relatively small number of patients and controls make meta-analysis and subgroup analysis challenging.

In summary, there appears to be an increase in proinflammatory cytokines most clearly for TNF-α, but probably also for IL-17 and IL-1β, in children with obsessive-compulsive and movement disorder symptoms compared to healthy controls. These cytokines can through their effect on the BBB give rise to neuroinflammation. This can potentially offer important insights into the pathogenesis of obsessive-compulsive and tic symptoms, as it implies that at least a subgroup of the affected patients could have an autoimmune pathogenesis. This could offer new treatment options for the afflicted children. However, more knowledge on the role of the immune system, including that of pro-inflammatory cytokines, is needed in the future. The knowledge from the existing studies is still limited and challenged by the heterogeneity of used methods and their relatively small sample size, and thus larger studies are needed to thoroughly examine the cytokine profile of children with obsessive-compulsive and movement disorder symptoms.

## Author contributions

RF conducted the review and authored the manuscript with supervision and revisions from CS, ND, and LS. All authors contributed to the article and approved the submitted version.

## Conflict of interest

The authors declare that the research was conducted in the absence of any commercial or financial relationships that could be construed as a potential conflict of interest. The handling editor UL-T declared a shared parent affiliation with the author ND at the time of review.

## Publisher's note

All claims expressed in this article are solely those of the authors and do not necessarily represent those of their affiliated organizations, or those of the publisher, the editors and the reviewers. Any product that may be evaluated in this article, or claim that may be made by its manufacturer, is not guaranteed or endorsed by the publisher.
